# Theoretical Study on the Photo-Oxidation and Photoreduction of an Azetidine Derivative as a Model of DNA Repair

**DOI:** 10.3390/molecules26102911

**Published:** 2021-05-14

**Authors:** Miriam Navarrete-Miguel, Antonio Francés-Monerris, Miguel A. Miranda, Virginie Lhiaubet-Vallet, Daniel Roca-Sanjuán

**Affiliations:** 1 Institut de Ciència Molecular, Universitat de València, 46071 València, Spain; Miriam.Navarrete@uv.es; 2 Departament de Química Física, Universitat de València, 46100 Burjassot, Spain; Antonio.Frances@uv.es; 3 Instituto Universitario Mixto de Tecnología Química UPV-CSIC, Universitat Politècnica de València, Consejo Superior de Investigaciones Científicas, Avda de los Naranjos s/n, 46022 Valencia, Spain; mmiranda@qim.upv.es (M.A.M.); lvirgini@itq.upv.es (V.L.-V.)

**Keywords:** azetidine, DNA repair, electron transfer, density functional theory, photochemistry, ring opening, redox properties

## Abstract

Photocycloreversion plays a central role in the study of the repair of DNA lesions, reverting them into the original pyrimidine nucleobases. Particularly, among the proposed mechanisms for the repair of DNA (6-4) photoproducts by photolyases, it has been suggested that it takes place through an intermediate characterized by a four-membered heterocyclic oxetane or azetidine ring, whose opening requires the reduction of the fused nucleobases. The specific role of this electron transfer step and its impact on the ring opening energetics remain to be understood. These processes are studied herein by means of quantum-chemical calculations on the two azetidine stereoisomers obtained from photocycloaddition between 6-azauracil and cyclohexene. First, we analyze the efficiency of the electron-transfer processes by computing the redox properties of the azetidine isomers as well as those of a series of aromatic photosensitizers acting as photoreductants and photo-oxidants. We find certain stereodifferentiation favoring oxidation of the *cis*-isomer, in agreement with previous experimental data. Second, we determine the reaction profiles of the ring-opening mechanism of the cationic, neutral, and anionic systems and assess their feasibility based on their energy barrier heights and the stability of the reactants and products. Results show that oxidation largely decreases the ring-opening energy barrier for both stereoisomers, even though the process is forecast as too slow to be competitive. Conversely, one-electron reduction dramatically facilitates the ring opening of the azetidine heterocycle. Considering the overall quantum-chemistry findings, *N*,*N*-dimethylaniline is proposed as an efficient photosensitizer to trigger the photoinduced cycloreversion of the DNA lesion model.

## 1. Introduction

The direct absorption of ultraviolet light causes the most abundant lesions in DNA, namely cyclobutane pyrimidine dimers (CPD) and (6-4) photoproducts (6-4PP) [1,2,3]. The impact of DNA lesions in living beings is mostly mitigated by DNA repair mechanisms that have been shaped throughout evolution. Regarding most living organisms (except in placental mammals), CPD and 6-4PP lesions can be repaired by photolyases, a class of enzymes that uses light to convert the dimeric pyrimidine lesions into the original monomers through the corresponding photoreactions [4,5,6]. Concerning the case of CPD, this repair mechanism takes place through an electron transfer to the lesion from the excited flavin adenine dinucleotide cofactor (FADH^−^), which is oriented toward the lesion, acting as an electron donor.

Photorepair of the 6-4PP is not as straightforward as for the CPDs, as migration of an OH or NH_2_ group from the 5′ to the 3´nucleobase is required to regenerate the native pyrimidines. Indeed, the involved processes have been a matter of debate and different mechanisms have been proposed along the years [5,6,7,8,9,10,11,12]. It long was believed that the (6-4) photolyase operates through an intramolecular dark rearrangement of the lesion, to form an oxetane/azetidine intermediate, which then acts as the electron-accepting species [13]. This hypothesis has been challenged on the basis of an in situ repair study of the crystallized photolyase containing a single lesion [7,8]. Nevertheless, the possible formation of an oxetane- or azetidine-like short-lived species has not been ruled out, and a more recent work has proposed an alternative mechanism involving a two-photon repair process with photogeneration as a reversible intermediate followed by photoinduced electron transfer [11]. The instability of the formed four-membered ring oxetane/azetidine intermediates is an important limitation because it prevents not only their isolation and characterization but also their use as substrates to investigate the electron-induced cycloreversion. Several models have been designed to mimic this intermediate to follow the electron-transfer processes by spectroscopic techniques [14,15] and theoretical methods [6,15]. Understanding DNA repair processes is crucial to comprehend not only life functioning and evolution on Earth, but also to develop applications that could contribute to the fight against the harmful biological consequences of unprotected exposure to solar radiation.

Regarding their chemical structure, oxetanes and azetidines are saturated four-membered-ring heterocyclic compounds containing an oxygen or nitrogen atom, respectively, with a reasonable chemical stability. While the former arise as plausible DNA lesions at thymine–thymine sequences, the latter are the analogues to oxetanes at thymine–cytosine sequences. Aside from their biological interest, these compounds also have been widely used in drug design. Overall, azetidines have not been studied to the same extent as oxetanes.

Furthermore, although DNA photoreduction has been mainly associated with photorepair, the significance of oxidative processes has long been proven in relation to the long-range charge transport [16,17,18]. Taking this context, the photoreactivity of azetidine intermediates is important to determine their photostability under photosensitization conditions and to evaluate the ability of some photo-oxidants to also act as photolyase mimics. Viewing a previous work, a model system of the azetidine intermediate obtained by the cycloaddition of thymine and 6-azauracil was designed [3]. A systematic study was also performed on the photo-oxidation and photoreduction by several photosensitizers (Phs) combining spectroscopy, electrochemistry, analytical tools, and multiconfigurational theoretical methods, revealing that photo-oxidation is more efficient in repairing this model [15]. 

**Figure 1:**
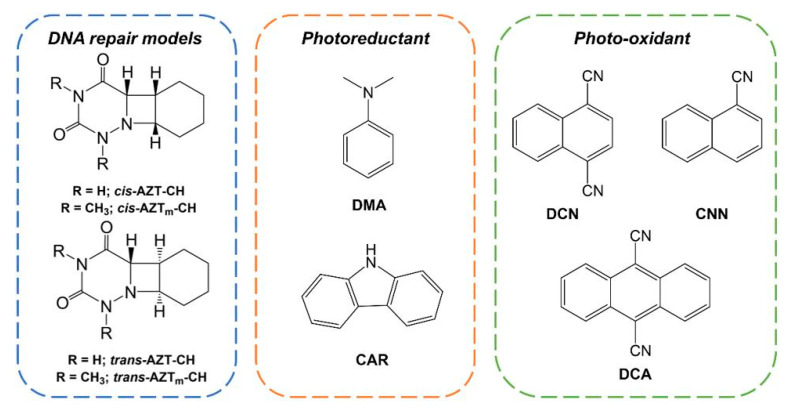
Structure of the *cis*- and *trans*- azetidine stereoisomers and the photosensitizers (Phs) studied in this work, classified according to their capacity to photo-oxidize or photoreduce the azetidine-cyclohexene model (AZT-CH) [19].

During this contribution, we consider two azetidine stereoisomers of another model system, namely *cis*- and *trans*-AZT_m_-CH (see Figure 1), synthesized through the [2+2] photocycloaddition of 6-aza-1,3-dimethyluracil and cyclohexene by Lhiaubet-Vallet and co-workers [19]. The capacity of *N*,*N*-dimethylaniline (DMA), carbazole (CAR) and several cyanoaromatics, 1,4-dicyanonaphthalene (DCN), 9,10-dicyanoanthracene (DCA) and 1-cyanonaphthalene (CNN) (Figure 1) to activate photoinduced electron transfer processes toward AZT_m_-CH was previously evaluated by means of steady-state and time-resolved fluorescence spectroscopy. The quenching kinetics revealed that AZT_m_-CH acts as an electron donor, i.e., reducing the Phs at different rates depending on the Phs, and that this process exhibits some stereoselectivity being more efficient for the *cis*-AZT_m_-CH stereoisomer. Cyclic voltammetry measurements were consistent with these trends [19]. It shall be noted that the opening of the azetidine ring could not be monitored in the experiments due to difficulties in detecting the cyclohexene that is formed. Thus, the objective of this work is double: first, to rationalize the molecular basis of the photo-oxidation and photoreduction processes already characterized by experimental techniques [19], and second, to study the ring opening processes subsequent to the electron transfer, whose experimental determination is highly challenging.

## 2. Results

Results are presented in two sections. The first section deals with the photoreductive and photo-oxidative properties of the demethylated AZT-CH stereoisomers and the different Phs. Here we will focus on the representative magnitudes to understand the electron-transfer processes. The second section presents the findings and discussion on the ring-opening mechanisms of the cationic and anionic systems in terms of the computed potential energy surfaces (PESs).

### 2.1. Photoreductive and Photo-Oxidative Properties

**Figure 2:**
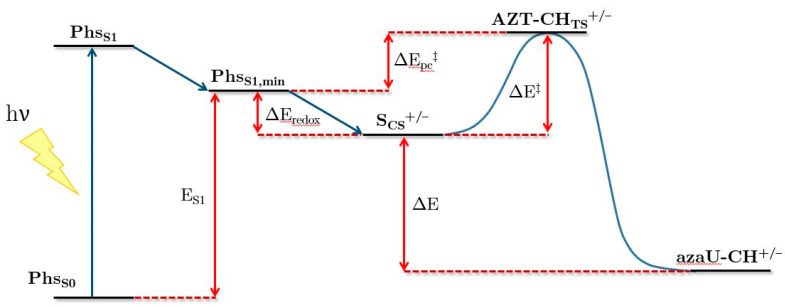
Scheme of the processes that take place during the ring-opening mechanism of the AZT-CH system. Phs_S0_ = photosensitizer in the ground state, Phs_S1_ = vertical absorption energy of the S_1_ state of the photosensitizer, Phs_S1,min_ = energy of the S_1_ state of the photosensitizer at its equilibrium geometry, S_CS_^+/−^ = charge separated state of the photosensitizer and the AZT-CH system, AZT-CH_TS_^+/−^ = transition state of the AZT-CH cationic or anionic state, azaU-CH^+/−^ = charge-separated reaction products, E_S1_ = adiabatic electronic transition energy for the S_1_ state of the photosensitizer, ΔE_redox_ = redox energy difference between AZT-CH and the photosensitizers in the excited state, ΔE^‡^ = activation energy, ΔE = energy difference between reactants and products in the charge separated state, ΔE_pc_^‡^ = overall ability of the Phs to induce the cycloreversion of AZT-CH.

Figure 2 represents a general scheme of the sequential processes that take place during the photoinduced electron transfer between the AZT-CH systems and the selected Phs (structures shown in Figure 1). The very first step is the excitation of the Phs by light absorption to the lowest-lying singlet state, Phs_S1_. Then, this state is assumed to relax to its minimum energy structure (Phs_S1,min_). Here, the representative magnitude is E_S1_, which corresponds to the adiabatic electronic transition energy. This magnitude can be related to the experimental singlet energy (E_S1,exp_). The excited state lives enough to trigger the electron transfer process to or from the AZT-CH stereoisomers (photoreduction or photo-oxidation, respectively), yielding the corresponding charge-separated state of the lesion, S_CS_^+/−^, i.e., an anion or a cation, and the corresponding charged Phs. The magnitude that describes the viability of this process is ΔE_redox_, indirectly connected to the bimolecular quenching rate, k_q_, obtained during the experiments. A more negative value of ΔE_redox_ is related to a higher rate. Subsequently, the ion pair can evolve opening the azetidine ring making use of the extra energy induced by the charge separation, which is in turn triggered by light absorption. ΔE^‡^ represents the height of the barrier for this sole process. The overall kinetics, nevertheless, is assessed considering also the energy of Phs_S1,min_ through the ΔE_pc_^‡^ magnitude, which is the energy gap between the corresponding transition state (AZT-CH_TS_^+/−^) and the Phs_S1,min_ energy. Once the final products, still with the corresponding charge separation, are obtained, the spontaneity of the reaction is ascertained through the energy difference between products and reactants, ΔE.

The first step to study the repair mechanism of the DNA lesion was to calculate the ionization potential (IP) and electron affinity (EA) of the azetidine derivative, in particular the *N*-demethylated compound AZT-CH, to determine the ability to lose or capture an electron, respectively. Density functional theory (DFT) (see Computational Details) with the M06-2X functional and the 6-31++G(d,p) basis set accurately describes the vertical IP (VIP) and adiabatic IP and adiabatic EA (AIP and AEA, respectively) magnitudes in these types of molecules, as validated by benchmark calculations on the thymine nucleobase using complete-active-space second-order perturbation theory (CASPT2) results as a reference [20,21]. The vertical magnitude in solution can be improved by strategies such as those defined by Slavicek, et al. beyond polarizable continuum model (PCM) [22,23]. Nevertheless, the important magnitudes here are the adiabatic ones. Regarding the case of vertical EA (VEA), where a temporary anion state is formed, its accurate description requires a different methodology [21,24,25], falling out from the scope of the present work.

IPs and EAs were obtained in the gas-phase and in acetonitrile solution for both *cis*- and *trans*-AZT-CH isomers. The values are reported in Table 1. The addition of the solvent stabilizes the anionic and cationic states, decreasing the IPs and increasing the EAs a few tenths of eV with respect to the gas-phase determinations. AIP can be indirectly related to the reduction potential of the AZT-CH, E_red,0,AZT-CH_. Note that a lower value of the IP of AZT-CH implies that it is easier to extract an electron, that is, to oxidize it, that is connected to a lower E_red,0,AZT-CH_. Likewise, a higher EA means a higher ability to take an electron, and then to be reduced. This is related to a higher value of the experimental E_red,0,AZT-CH_. During the experiments, only the oxidation of the AZT-CH was observed. Focusing on the VIP and AIP values, they agree with the experiments performed in [19], thus supporting stereopreference for the oxidation of the *cis*- isomer as compared to the *trans*- one.

The AIP and AEA of the selected Phs were also calculated, as well as the E_S1_ and the energy changes in the photoreduction and photo-oxidation reactions (Phs_red_* + AZT-CH → Phs_red_^•^^+^ + AZT-CH^•^^−^ and Phs_ox_* + AZT-CH → Phs_ox_^•^^−^ + AZT-CH^•^^+^, respectively). All these values are reported in Table 2, together with the experimental E_S1_ (E_S1,exp_) [19] and the experimental reduction potential in the ground state, E_red,0,Phs_ [26]. When comparing the computed E_S1_ with E_S1,exp_, the photoreductants exhibit larger experimental/theoretical discrepancies compared to the photo-oxidants. Nevertheless, both data sets follow the same trend, DMA having the highest E_S1_, whereas DCA has the lowest value. 

DCN and DCA have similar and less negative E_red,0,Phs_ than CNN, which implies a higher ability to accept one electron from the AZT-CH in the photo-oxidation process. This trend is captured also in the computed AEA, which is clearly lower for CNN. Discrepancies between AEA and E_red,0,Phs_ for DCN and DCA can be due to experimental factors or theory/experimental inability to discriminate between such similar redox properties. Regarding ΔE_redox_, on the one hand, it can be seen that within the photoreductants, DMA shows a negative value for both isomers, while CAR has a positive value instead. These values indicate that the photoreduction of the AZT-CH by the former Phs is thermodynamically favorable, while the photoreduction by CAR is not spontaneous. This coincides with the experimental findings reported in [19], where the authors did not observe any quenching of CAR fluorescence, whereas DMA indeed exhibited slight activity in the fluorescence experiments. It is worth mentioning that DMA has a short excited-state lifetime, so a very high quenching constant is necessary to observe its deactivation. Conversely, concerning the photo-oxidants, DCN shows the most negative value of ΔE_redox_ for both AZT-CH stereoisomers, being the best photo-oxidant Phs, in agreement with the highest k_q_ value obtained during the experiments. Regarding DCA and CNN, the process is less efficient as their values are closer to zero.

Overall, Table 2 shows that photo-oxidation is favorable for the three cyanoaromatic Phs, namely DCN, CNN, and DCA (Figure 1), whereas photoreduction is only favorable by DMA. The stereopreference for the *cis*-AZT-CH isomer in the photo-oxidation process is maintained for all the Phs, in agreement with the experimental determinations.

### 2.2. Ring-Opening Mechanisms of cis- and trans-AZT-CH Radical Anion and Cation

The chemical mechanism of the photorepair of the AZT-CH model lesion to obtain the separated monomers is analyzed herein in terms of the corresponding energy profiles obtained with appropriate computational strategies (see Computational Details).

The ring opening of the neutral *cis*- and *trans*- isomers corresponds to the reaction without any electron transfer, i.e., a non-catalyzed reaction. It proceeds through a concerted mechanism in which the C-C and C-N bond breaking is characterized by a single transition state (TS), as shown in Figures S1 and S2 for both *cis*- and *trans*- isomers, respectively, and Table S1 of the Supporting Information. The reaction is exergonic (negative ΔE); however, the activation energy (ΔE^‡^) is extremely high, about 65 kcal mol^−1^, indicating extremely slow kinetics and, thus, unambiguously classifying the process as thermally prohibited. 

**Figure 3:**
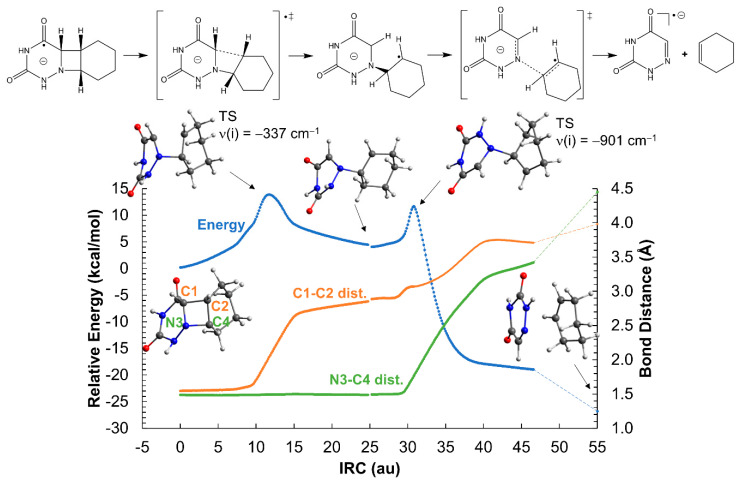
Opening of the azetidine ring of the *cis*-AZT-CH system in the gas-phase. The reaction profile corresponds to the reduced system with a net charge of −1 and doublet multiplicity. ‡ indicates transition state.

**Figure 4:**
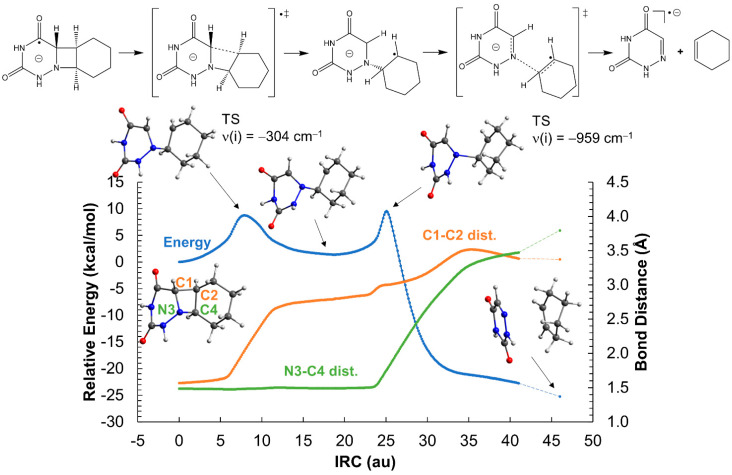
Opening of the azetidine ring of the *trans*-AZT-CH system in the gas-phase. The reaction profile corresponds to the reduced system with a net charge of −1 and doublet multiplicity. ‡ indicates transition state.

Contrary to the neutral system, the ionic azetidine ring opens in two steps corresponding to the C_1_-C_2_ and N_3_-C_4_ bond scissions, respectively, with clearly smaller activation energies. The addition of an extra electron to the azetidine ring weakens the C-C σ bond of *cis*-AZT-CH, decreasing the activation energy of the C_1_-C_2_ cleavage to ca. 14 kcal mol^−1^ (see Figure 3). Regarding the *trans*- isomer, the activation energy is even lower, about 9 kcal mol^−1^, as displayed in Figure 4. The process yields an intermediate with a relative energy close to 0 kcal mol^−1^, in which the two monomers are bound only through a single N_3_-C_4_ connection. This species can evolve with almost equal probability, either breaking the N_3_-C_4_ bond, thereby producing the products and releasing a large quantity of energy >20 kcal mol^−1^, or reforming the C_1_-C_2_ bond to regenerate the azetidine ring. The formation of the products can be considered, however, kinetically irreversible.

Analysis of the Mulliken charges and spin densities (Tables S2, S4, and S5) reveal that the negative charge is localized over the 6-azauracil fragment for both *cis*- and *trans*-AZT-CH throughout the reaction. Figure 3 and Figure 4 show schematic representations of the charge and spin densities that fit the most important values determined with DFT. Surprisingly, the system exhibits a certain charge-transfer character at the reactants region, in which the cyclohexene subsystem has a positive charge (e.g., 0.45 for the *cis*- stereoisomer) and 6-azauracil has a negative charge greater than −1 (−1.45). This striking charge separation decreases along the ring-opening reaction and, at the products region, the negative charge and the unpaired electron is localized exclusively over 6-azauracil.

An alternative ring aperture mechanism in which the N_3_-C_4_ bond breaks prior to the C_1_-C_2_ cleavage has been explored in Figures S3 and S4. Regarding both *cis*- and *trans*-AZT-CH stereoisomers, the N_3_-C_4_ scission yields an intermediate with a relative energy close to zero; however, the energy barrier heights are significantly higher than that of the C_1_-C_2_ bond breaking shown in Figure 3 and Figure 4. Particularly, the initial N_3_-C_4_ ruptures have energy penalties of ~21 and ~22 kcal mol^−1^ for the *cis*- and *trans*-AZT-CH anionic systems, respectively, clearly higher than the initial C_1_-C_2_ breaks mentioned above (~14 and ~9 kcal mol^−1^ for the *cis*- and *trans*-AZT-CH, respectively). Hence, it can be safely concluded that, for the anionic AZT-CH systems, the C_1_-C_2_ bond breaking initiates the ring aperture, since the N_3_-C_4_ scission is deemed much slower.

**Figure 5:**
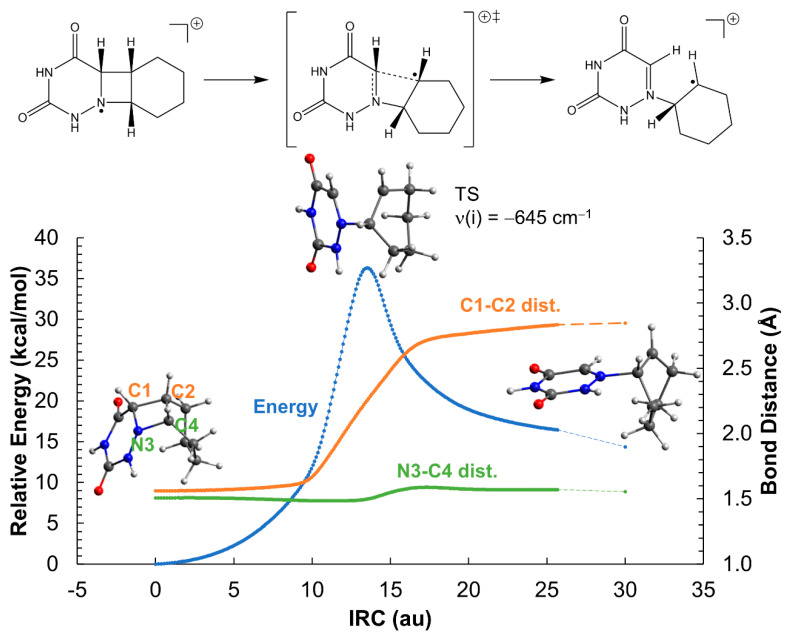
Opening of the azetidine ring of the *cis*-AZT-CH system in the gas-phase. The reaction profile corresponds to the oxidized system with a net charge of +1 and doublet multiplicity. Only the C_1_-C_2_ bond break is shown, the N_3_-C_4_ bond cleavage is displayed in a different figure and is common to both *cis*- and *trans*- isomers. ‡ indicates transition state.

**Figure 6:**
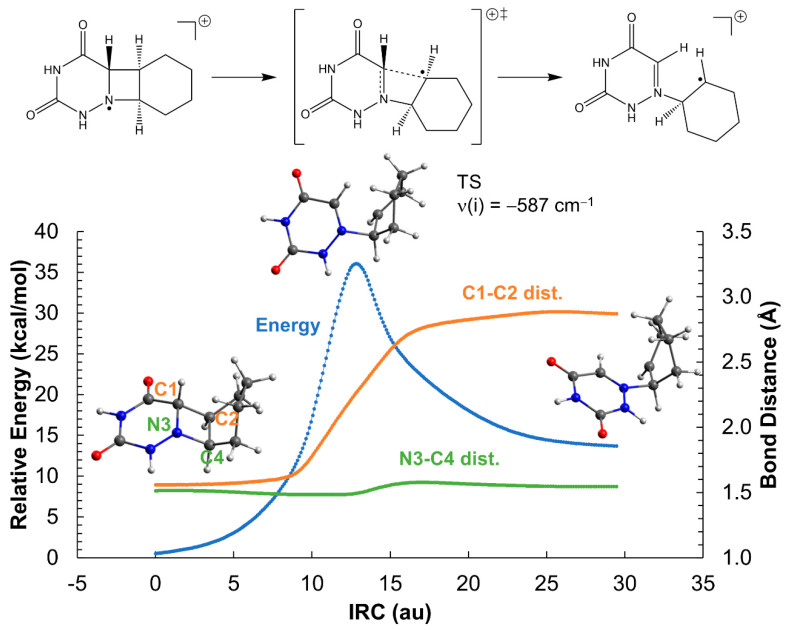
Opening of the azetidine ring of the *trans*-AZT-CH system in the gas-phase. The reaction profile corresponds to the oxidized system with a net charge of +1 and doublet multiplicity. Only the C_1_-C_2_ bond break is shown, the N_3_-C_4_ bond cleavage is displayed in a different figure and is common to both *cis*- and *trans*- isomers. ‡ indicates transition state.

The scenario is different for the cationic AZT-CH molecules. The C_1_-C_2_ bond breaking has an activation energy of 36 kcal mol^−1^ for both *cis*- and *trans*-AZT-CH (see Figure 5 and Figure 6, respectively), which is much larger than that of the anionic isomers mentioned above, although smaller than that of the neutral system. Again, an intermediate in which both 6-azauracil and cyclohexene rings are connected solely through the N_3_-C_4_ bond is formed, although much more unstable (relative energy ~15 kcal mol^−1^) than the anionic analogue.

**Figure 7:**
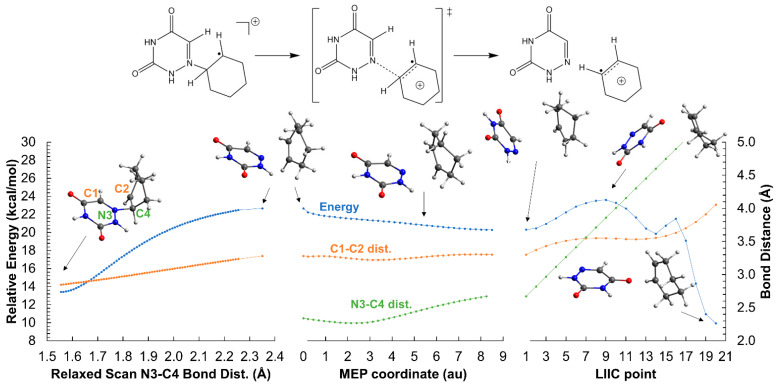
N_3_-C_4_ scission of the AZT-CH system in the gas-phase. Relaxed scan of the N_3_-C_4_ coordinate (**left** hand side), minimum energy path (MEP, **center**) from the highest-energy point of the scan, and linear interpolation of internal coordinates (LIIC, **right** hand side) between the last point of the MEP and the optimized products. The reaction profile corresponds to the oxidized system with a net charge of +1 and doublet multiplicity. The N_3_-C_4_ bond cleavage is common to both *cis*- and *trans*- isomers, and the transition state-like structure corresponds to a N_3_-C_4_ distance of 2.35 Å. Energies are relative to the *trans*-AZT-CH cation. ‡ indicates transition state.

The second step in the ring-opening mechanism consists of the N_3_-C_4_ bond breaking and is common to both isomers (see Figure 7). This part of the mechanism was obtained by means of scan, minimum energy path (MEP) and linear interpolation of internal coordinates (LIIC) computational strategies, and obtaining an upper-bound for the energy barrier height of ~10 kcal mol^−1^ from the intermediate species. Overall, the formation of the cationic products is endergonic with respect to the reactants, indicating that the reverse reaction is equally or even more competitive than the forward process. Consequently, an irreversible aperture of the azetidine ring would require a back electron transfer to generate the neutral 6-azauracil and cyclohexene (and, of course, the corresponding neutral Phs) to trap the products. Interestingly, the cationic π-stacking interaction between the 6-azauracil ring and the C_2_=C_4_ bond of cyclohexene is poor, as the system interacts via H-bonding instead (right-hand side of Figure 7).

Analysis of the Mulliken charges and spin densities (Tables S3, S6, and S7) indicate that the positive charge is localized mostly over the cyclohexene fragment, although at the reactant region one third or one fifth (depending on the AZT-CH stereoisomer) is localized over 6-azauracil. The charge is less shared as the reaction proceeds, and at the product region 90% of the charge is localized only over cyclohexene. This is in agreement with the lower ionization energy of cyclohexene as compared to 6-azauracil measured experimentally by photoelectron spectroscopy [27,28].

**Figure 8:**
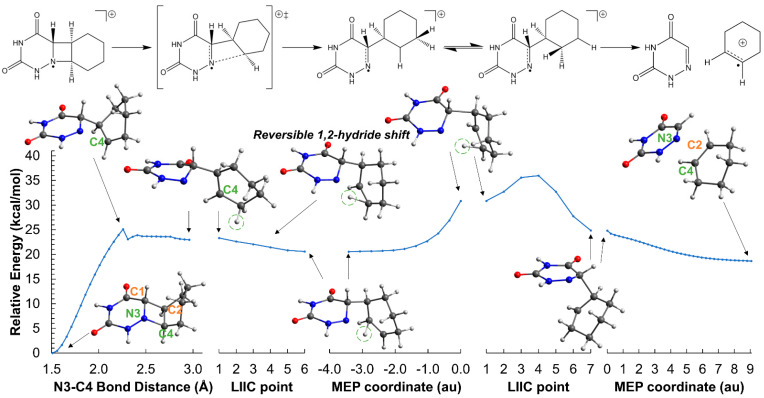
Opening of the azetidine ring of the *trans*-AZT-CH system in the gas-phase initiated by the N_3_-C_4_ bond breaking. The reaction profiles correspond to the oxidized system with a net charge of +1 and doublet multiplicity, and have been obtained through relaxed scans of the N_3_-C_4_ bond distance, LIIC between relevant structures, and MEP determinations. The initial points for both MEP profiles were computed through relaxed scan calculations of the C_1_-C_2_ bond distance freezing the N_3_-C_4_ coordinate at 2.959 Å to avoid the return of the system to the reagents region. The hydrogen atom that undergoes the 1,2-hydride shift is highlighted with green dashed circles. The pathway that connects the last MEP structure to the products minimum shown in Figure 7 has not been computed. ‡ indicates transition state.

The ring aperture mediated by the initial N_3_-C_4_ bond breaking also has been explored for the cationic AZT-CH system. The energy profiles are shown in Figure 8, Figures S5 and S6. Overall, the energy barrier heights are higher or similar to that of the ring opening initially activated by the C_1_-C_2_ bond cleavage, even though the profiles for the former reactions must be considered as upper bounds of the actual energy barrier heights, as no TSs were converged due to complexities of the PESs. Instead, more approximated computational methods, such as relaxed scans and coordinate interpolations, were adopted which, in combination with MEP determinations, provide good estimations for the ring apertures.

The comparison between the two reaction mechanisms, i.e., ring apertures initiated by either the C_1_-C_2_ or the N_3_-C_4_ bond breaking, reveal that the highest energy penalty is associated with the cleavage of the C_1_-C_2_ bond. Interestingly, the *trans*-AZT-CH cation exhibits 1,2-hydride shifts typical of carbocations (see Figure 8), a process that has not been observed in the *cis*-AZT-CH stereoisomer due to the different hydrogen disposition and relative arrangements of the 6-azauracil and cyclohexene moieties. Globally, these 1,2-rearrangements are reversible and only stabilize the energy of the intermediate without changing the molecular mechanism of the reaction. Actually, the 1,2-hydride shift in the C_2_-C_4_ position leads to a species whose C_1_-C_2_ bond scission is much more energetic (see Figure S6). Considering these results, it is reasonable to think that the ring opening reaction triggered by the initial N_3_-C_4_ cleavage is probably more efficient for the *trans*-AZT-CH cation, even though the energy barriers (>30 kcal mol^−1^) are still large and predict slow ring-opening reactions with the photo-oxidants considered in this work.

The impact of the solvation and the influence of the temperature and entropic effects on the kinetics and thermodynamics of the ring-opening reactions can be assessed with the data compiled in Table 3 and Table 4, referring to the electronic thermodynamics (ΔE), the electronic activation energy (ΔE^‡^), their respective corrected values with the zero-point vibrational energy (ZPVE), i.e., ΔE_0_ and ΔE_0_^‡^, and the Gibbs free energies (ΔG and ΔG^‡^). Note that the DFT protocol used here was previously validated by comparing to data generated at the CASPT2 level [15]. Commented above, the ΔE values indicate that both isomers undergo a spontaneous ring-opening reaction in the anionic state, while the same process in the cationic state is clearly endergonic. The energetics in solution show changes of a few kcal mol^−1^ with respect to those obtained in the gas-phase, consistent with the expected variations of the dipole moment modules as the ring aperture proceeds. These changes do not alter the interpretations based on the gas-phase results in any case. It also can be observed that the ZPVE correction decreases the activation barriers and increases the thermodynamic stability of the products, evidencing a preferential stabilization of the TSs and the separated monomers, as compared to the reactants (four-membered azetidine ring). Unlike in the electron-transfer processes, where a stereopreference for the *cis*-AZT-CH isomer is observed, the ring-opening mechanisms show no preference for any of the isomers.

Finally, the global ability of the Phs to induce the photocycloreversion reaction of both isomers can be ascertained through the ΔE_pc_^‡^ magnitude, which considers both the electron-transfer efficiency and the kinetics of the ring cleavage. The values are reported in Table 5. The photocycloreversion through oxidation mediated by DCA, DCN, and CNN is ~4–5 kcal mol^−1^ more favorable for the *cis*-AZT-CH system, even though all three ΔE_pc_^‡^ values lie above 20 kcal mol^−1^ and, therefore, are deemed slow. Regarding the photocycloreversion through reduction, different behaviors are found. Regarding CAR, the overall process is not favorable either. Conversely, in DMA, the computed data yield of ΔE_pc_^‡^ is close to zero, thus indicating that the TS for the ring opening is almost energetically degenerated with the Phs_S1,min_ structure (see Figure 2), being clearly the most favorable process studied in this work. Strikingly, the ΔE_pc_^‡^ values for the photocycloreversion through reduction invert the stereoselectivity, being in these cases more favorable for the *trans*- isomer. 

## 3. Computational Details

The DFT method as implemented in the Gaussian 09 (D.01 revision) software package [29] was employed in this study. All geometry optimizations, frequency calculations, IP and EA determinations of the Phs and AZT-CH systems were conducted using the Minnesota DFT/M06-2X hybrid functional [30] and the 6-31++G** basis set. This computational method provides suitable descriptions of the reactivity of similar DNA-based systems [15,31,32,33,34]. The true nature of the stationary points was checked by analyzing the sign of the vibrational normal modes (all positive for minima and only one negative for TSs). Solvent effects (acetonitrile) were included by means of the polarizable continuum model (PCM) using the default Gaussian 09 settings. The PCM-M06-2X method was applied on top of the converged M06-2X geometries in the gas-phase, allowing a clear and systematic evaluation of the solvent effects by comparing both gas-phase and solution energetics. Whereas the experiments on the model system are carried out in solution [19], in more hydrophobic situations (such as the DNA double strand) the gas-phase results presented in this work also might be informative, representing an additional advantage of this computational protocol. The small energy differences found between the gas-phase and PCM determinations (<5 kcal mol^−1^) and previous works on related systems [35] suggest a very small or negligible influence of the solvent in the optimizations.

TS optimizations followed by intrinsic reaction coordinate (IRC) calculations were conducted to obtain the full ring-opening profiles of the *cis*- and *trans*-AZT-CH anion and neutral systems. The last points of the IRC were optimized without any constraint to fully minimize the systems. Regarding the case of the cation, the IRC strategy only was used to describe the C_1_-C_2_ bond breaking because the TS related to the N_3_-C_4_ bond breaking was not found. Consequently, the bond breaking was explored through relaxed scans followed by a MEP determination from the highest-energy point found through the scan exploration, achieving the ring opening even though falling into a planar region far from the real minimum of the products. The profile between the last point of the MEP and the optimized products was obtained through linear interpolations (LIIC), thus estimating an upper bound for the energy barrier.

## 4. Conclusions

The photoinduced cycloreversion processes through photoreduction and photo-oxidation reactions of the AZT-CH molecule were studied in this work by means of quantum chemistry. The photo-reductive and photo-oxidative properties of this system and those of distinct photosensitizers were evaluated in terms of the S_1_ vertical and adiabatic energies, electronic affinities, and ionization potentials, finding a stereoselectivity for the *cis*-AZT-CH isomer consistent with the experimental results reported in reference [19]. Regarding the ring-opening mechanisms of the cationic and anionic systems, the obtained results indicate that both processes proceed through stepwise mechanisms, in which the C_1_-C_2_ and N_3_-C_4_ bonds break in different steps. Regarding the AZT-CH anion, the initial C_1_-C_2_ rupture is unambiguously more favorable, whereas for the AZT-CH cation, both initial C_1_-C_2_ and N_3_-C_4_ ruptures have similar energy barrier heights, in which the rupture of the former bond has the largest penalty. The cationic reaction requires much higher activation energies as compared to the anionic systems. No significant stereopreference is observed during these processes. Overall, considering both electron-transfer feasibility and ring-opening energetics, it can be safely concluded that the photocycloreversion through reduction mediated by the photosensitizer DMA is the most favorable process studied in this work. 

## Figures and Tables

**Table 1:** Vertical and adiabatic ionization potentials (VIPs, AIPs) and adiabatic electron affinities (AEAs) in eV (kcal mol^−1^ within parentheses) for *cis*- and *trans*-AZT-CH isomers in the gas phase and in acetonitrile. The values were computed with the density functional theory DFT/M06-2X method, the 6-31++G(d,p) basis set, and the polarizable continuum model (PCM) approach for the solvent. The experimental reduction potentials vs. SCE (Saturated Calomel Electrode) (E_red,0,AZT-CH_, in V) for *cis*- and *trans*-AZT_m_-CH isomers (see Figure 1) also are shown [19].

	VIP	AIP	AEA	E_red,0,AZT-CH_
*cis*-AZT-CH				
gas-phase	8.97 (206.8)	8.11 (186.9)	−0.48 (−11.0)	-
Acetonitrile	6.98 (160.9)	6.12 (141.0)	1.55 (35.8)	1.26
*trans*-AZT-CH				
gas phase	9.28 (214.0)	8.28 (190.8)	−0.61 (−14.0)	-
Acetonitrile	7.28 (167.9)	6.26 (144.4)	1.58 (36.4)	1.51

**Table 2:** Adiabatic absorption energies of the lowest-lying singlet excited state (E_S1_), experimental E_S1_ (E_S1,exp_) [19], AIPs or AEAs (AIP/AEA) of the selected Phs, energy changes (ΔE_redox_) related to the photoreduction or photo-oxidation processes in eV (kcal mol^−1^ within parentheses) computed for the *cis*- and *trans*-isomers in acetonitrile using the DFT/M06-2X method and the 6-31++G(d,p) basis set in acetonitrile solution (PCM), experimental ground state reduction potentials [26] (E_red,0,Phs_) in V, and the experimental bimolecular quenching rate (k_q_) for the *cis*- and *trans*-isomers, expressed in 10^9^ M^−1^·s^−1^.

	E_S1_	E_S1,exp_	AIP/AEA	E_red,0,Phs_	*cis*-ΔE_redox_	*trans*-ΔE_redox_	*cis*-k_q_	*trans*-k_q_
Photoreduction								
DMA	4.39 (101.1)	3.76 (86.7)	5.41 (124.7)	0.68	−0.53 (−12.3)	−0.56 (−12.9)	N.D.	N.D.
CAR	4.20 (96.8)	3.50 (80.7)	6.02 (138.9)	0.96	0.27 (6.2)	0.24 (5.6)	N.D.	N.D.
Photo-oxidation								
DCA	2.74 (63.3)	2.86 (66.0)	3.57 (82.2)	−1.0	−0.19 (−4.4)	−0.05 (−1.1)	10	7.7
DCN	3.62 (83.5)	3.75 (86.5)	3.14 (72.5)	−0.93	−0.65 (−15.0)	−0.51 (−11.6)	6.4	4.6
CNN	3.95 (91.1)	3.88 (89.5)	2.43 (56.0)	−2.21	−0.26 (−6.0)	−0.12 (−2.7)	3.2	2.6

**Table 3:** Energy differences between products and reactants (ΔE, ΔE_0_, ΔG) and activation energies (ΔE^‡^, ΔE_0_^‡^, ΔG^‡^) for the ring-opening aperture of the *cis*-AZT-CH radical anion and cation, given in kcal mol^−1^.

Methodology	*cis*-AZT-CH^•−^		*cis*-AZT-CH^•+^	
	ΔE	ΔE^‡^	ΔE	ΔE^‡^
M06-2X	−26.79	13.90	10.89	36.32
PCM-M06-2X	−24.74	16.21	14.67	35.03
	ΔE_0_	ΔE_0_^‡^	ΔE_0_	ΔE_0_^‡^
M06-2X	−29.22	12.02	7.06	33.84
PCM-M06-2X	−27.05	14.45	10.47	32.18
	ΔG	ΔG^‡^	ΔG	ΔG^‡^
M06-2X	−32.21	11.38	2.63	33.40
PCM-M06-2X	−30.16	13.69	6.40	32.11

**Table 4:** Energy differences between products and reactants (ΔE, ΔE_0_, ΔG) and activation energies (ΔE^‡^, ΔE_0_^‡^, ΔG^‡^) for the ring-opening aperture of the *trans*-AZT-CH radical anion and cation, given in kcal mol^−1^.

Methodology	*trans*-AZT-CH^•−^		*trans*-AZT-CH^•+^	
	ΔE	ΔE^‡^	ΔE	ΔE^‡^
M06-2X	−25.23	8.78	9.93	36.08
PCM-M06-2X	−20.80	13.68	14.81	36.16
	ΔE_0_	ΔE_0_^‡^	ΔE_0_	ΔE_0_^‡^
M06-2X	−27.65	6.85	6.36	33.85
PCM-M06-2X	−23.19	11.77	11.02	33.71
	ΔG	ΔG^‡^	ΔG	ΔG^‡^
M06-2X	−30.28	6.75	2.39	33.64
PCM-M06-2X	−25.84	11.65	7.28	33.72

**Table 5:** Overall energy barrier height (ΔE_pc_^‡^) computed in acetonitrile for the ring opening of the *cis*- and *trans*-AZT-CH isomers by photoreduction (Phs_red_* + AZT-CH → Phs_red_^•^^+^ + azaU-CH^•^^−^) and photo-oxidation (Phs_ox_* + AZT-CH → Phs_ox_^•^^−^ + AZT-CH^•^^+^), with Phs_red_ = DMA, CAR and Phs_ox_ = DCA, DCN, CNN. Energies are given in kcal mol^−1^.

	ΔE_pc_^‡^ *cis*-AZT-CH	ΔE_pc_^‡^ *trans*-AZT-CH
CAR	22.4	19.3
DMA	4.0	0.8
DCA	30.6	35.1
DCN	20.1	24.5
CNN	29.0	33.5

## Data Availability

The data presented in this study are available on request from the corresponding author.

## Supplementary Materials

The following are available online. Figures S1 and S2: ring-opening reaction profile for the neutral AZT-CH, Figures S3 and S4: ring-opening reaction profile for the anionic AZT-CH system initiated by N_3_-C_4_ bond breaking. Figures S5 and S6: ring-opening reaction profile for the cationic AZT-CH system initiated by N_3_-C_4_ bond breaking. Figure S7: full atom labelling of AZT-CH, Table S1: Thermodynamic and kinetic parameters of the neutral AZT-CH ring aperture reaction, Tables S2 and S3: Mulliken atomic spin densities, Tables S4–S7: Mulliken atomic charges.

## Sample Availability

Samples of the compounds are not available from the authors.
