# Supplementary material for: Theoretical Study on the Photo-Oxidation and Photoreduction of an Azetidine Derivative as a Model of DNA Repair
Source: Molecules. 2021 May 14;26(10):2911. doi: 10.3390/molecules26102911 (PMC8157190; doi:10.3390/molecules26102911)
Supplement: Supplementary file 1 [file molecules-26-02911-s001.zip › molecules-1198303-supplementary.pdf]

Supporting Information for

Theoretical Study on the Photo-oxidation and Photoreduction of an Azetidine Derivative as a Model of DNA Repair

Miriam Navarrete-Miguel[1], Antonio Francés-Monerris[2], Miguel A. Miranda[3], Virginie Lhiaubet-Vallet[3] and Daniel Roca-Sanjuán[1,*]

[1] Institut de Ciència Molecular, Universitat de València, València 46071, Spain; Miriam.Navarrete@uv.es
[2] Departament de Química Física, Universitat de València, 46100 Burjassot, Spain; Antonio.Frances@uv.es
[3] Instituto Universitario Mixto de Tecnologia Química UPV-CSIC, Universitat Politècnica de València, Consejo Superior de Investigaciones Científicas, Avda de los Naranjos s/n, 46022 Valencia, Spain. e-mail: mmiranda@qim.upv.es, lvirgini@itq.upv.es
* Correspondence: Daniel.Roca@uv.es

Table of contents

Figure S1. Opening of the azetidine ring of the *cis*-AZT-CH system in the gas phase. The reaction profile corresponds to the neutral system.

Figure S2. Opening of the azetidine ring of the *trans*-AZT-CH system in the gas phase. The reaction profile corresponds to the neutral system.

Table S1. Energy differences between products and reactants (ΔE, ΔE_0, ΔG) and activation energies ($\Delta E^{‡}$, $\Delta E_0^{‡}$, $\Delta G^{‡}$) for the photocycloreversion of the neutral *cis*- and *trans*-AZT-CH isomers, in the gas phase and in solution. Energies are given in kcal mol^{-1}.

Figure S3. Opening of the azetidine ring of the *cis*-AZT-CH system in the gas phase initiated by the N_3-C_4 bond breaking. The reaction profile corresponds to the reduced system with a net charge of -1 and doublet multiplicity.

Figure S4. Opening of the azetidine ring of the *trans*-AZT-CH system in the gas phase initiated by the N_3-C_4 bond breaking. The reaction profile corresponds to the reduced system with a net charge of -1 and doublet multiplicity.

Figure S5. Opening of the azetidine ring of the *cis*-AZT-CH system in the gas phase initiated by the N_3-C_4 bond breaking. The reaction profiles correspond to the oxidized system with a net charge of +1 and doublet multiplicity, and have been obtained through relaxed scans of the N3-C4 and C1-C2 bond distances, linear interpolation of internal coordinates (LIIC), and MEP determinations. As indicated by the back arrows, the relaxed scan of the C_1-C_2 bond distance was initiated from the most stable structure that exhibit N_3-C_4 bond cleavage (2.808 Å), and performed freezing the N_3-C_4 bond distance at this value to avoid the return of the system to the reagents region. The pathway that connects the last MEP structure to the products minimum shown in Figure 7 of the main text has not been computed.

Figure S6. Opening of the azetidine ring of the *trans*-AZT-CH system in the gas phase initiated by the N_3-C_4 bond breaking. The reaction profiles correspond to the oxidized system with a net charge of +1 and doublet multiplicity, and have been obtained through relaxed scans of the N_3-C_4 and C_1-C_2 bond distances and linear interpolation of internal coordinates (LIIC) between relevant structures. LIIC point number 14 has been optimized without any constraint. The hydrogen atom that undergoes the 1,2-hydride shift is highlighted with green dashed circles.

Figure S7. Atom labels of the AZT-CH system.

Table S2. Mulliken atomic spin densities for the relevant points on the ring opening reaction of *cis*- and *trans*-AZT-CH isomers of the anionic system. Only the most relevant positive atomic densities are shown, the total spin density is 1.

Table S3. Mulliken atomic spin densities for the relevant points on the ring opening reaction of *cis*- and *trans*-AZT-CH isomers of the cationic system. Only the most relevant positive atomic densities are shown, the total spin density is 1.

Table S4. Mulliken atomic charges for each atom of the cis-AZT-CH anion and sum of the charges for both fragments, *i.e.* 6-azauracil and cyclohexene in the reactants, transition states and products.

Table S5. Mulliken atomic charges for each atom of the *trans*-AZT-CH anion and sum of the charges for both fragments, *i.e.* 6-azauracil and cyclohexene in the reactants, transition states and products.

Table S6. Mulliken atomic charges for each atom of the *cis*-AZT-CH cation and sum of the charges for both fragments, *i.e.* 6-azauracil and cyclohexene, in the reactants, transition states and products.

Table S7. Mulliken atomic charges for each atom of the *trans*-AZT-CH cation and sum of the charges for both fragments, *i.e.* 6-azauracil and cyclohexene, in the reactants, transition states and products.

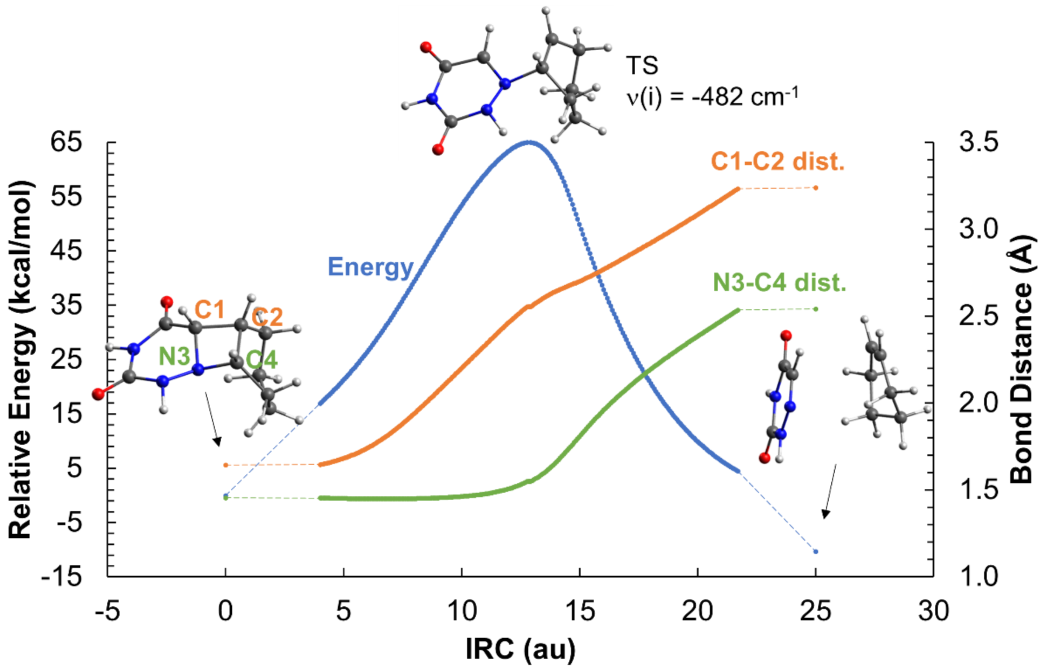

Figure S1. Opening of the azetidine ring of the *cis*-AZT-CH system in the gas phase. The reaction profile corresponds to the neutral system.

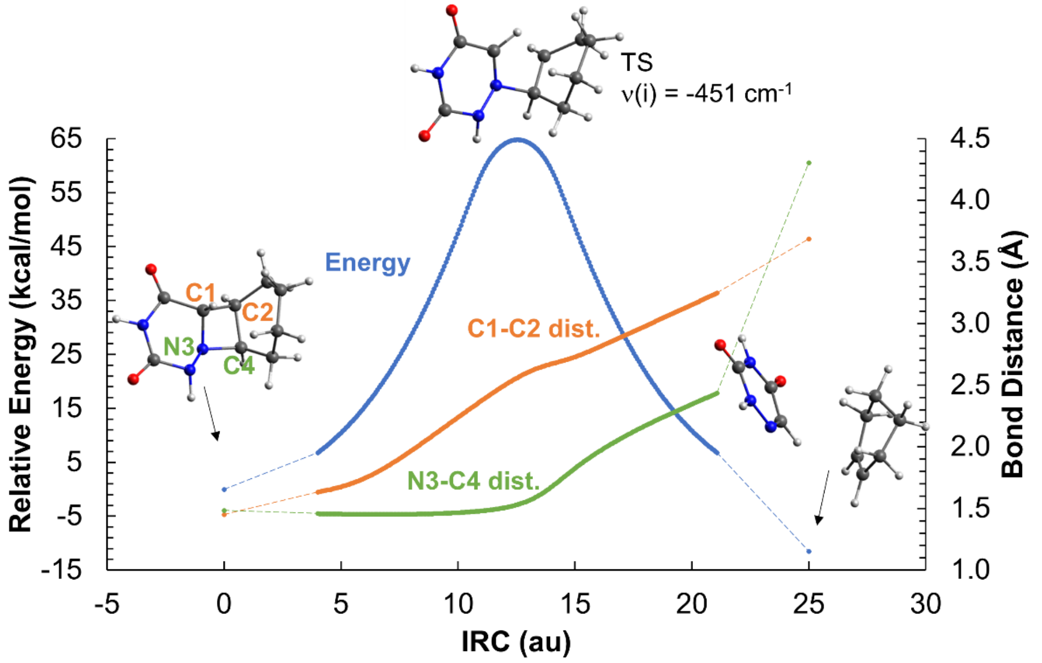

Figure S2. Opening of the azetidine ring of the *trans*-AZT-CH system in the gas phase. The reaction profile corresponds to the neutral system.

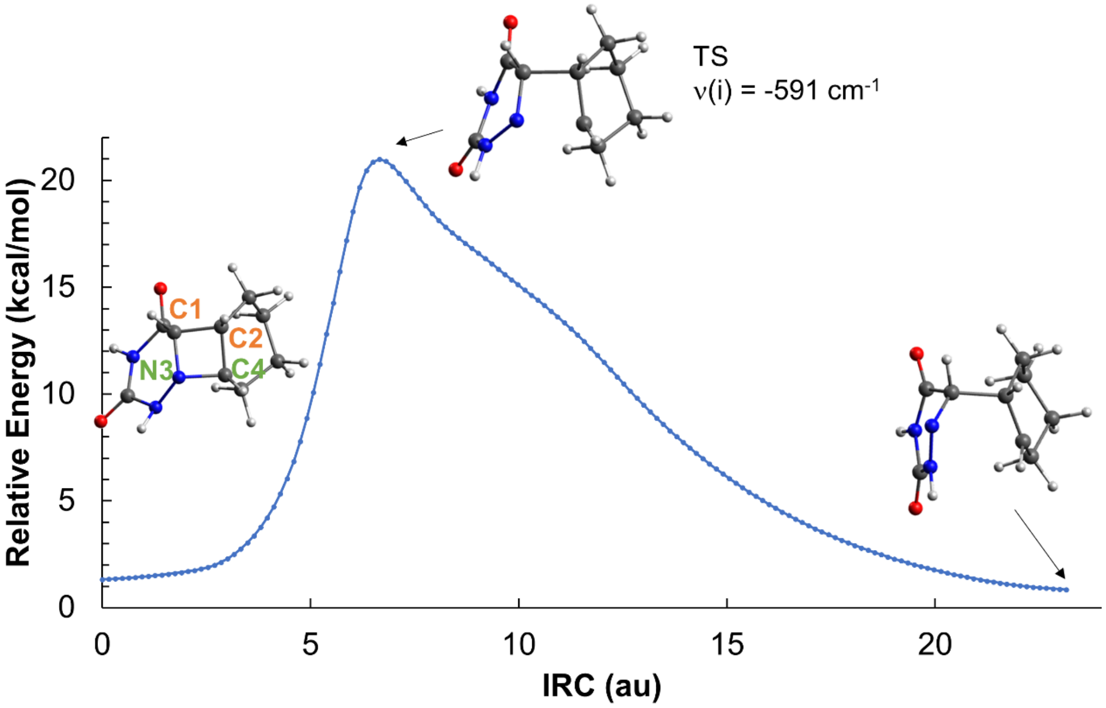

Figure S3. Opening of the azetidine ring of the *cis*-AZT-CH system in the gas phase initiated by the N_3-C_4 bond breaking. The reaction profile corresponds to the reduced system with a net charge of -1 and doublet multiplicity.

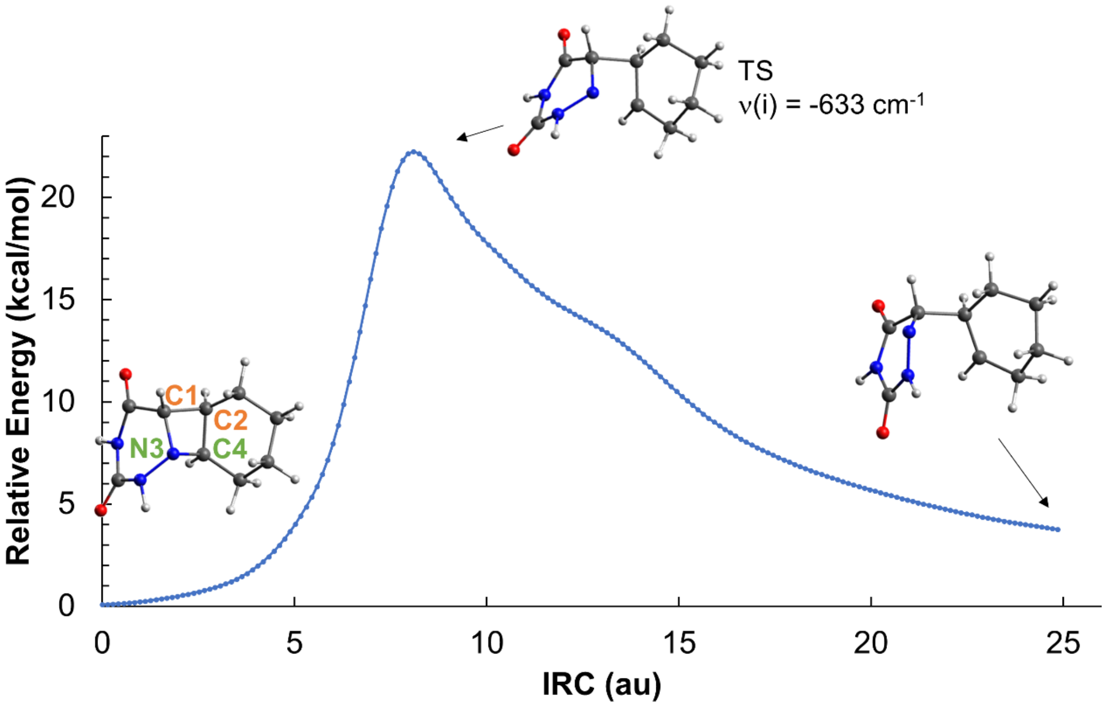

Figure S4. Opening of the azetidine ring of the *trans*-AZT-CH system in the gas phase initiated by the N_3-C_4 bond breaking. The reaction profile corresponds to the reduced system with a net charge of -1 and doublet multiplicity.

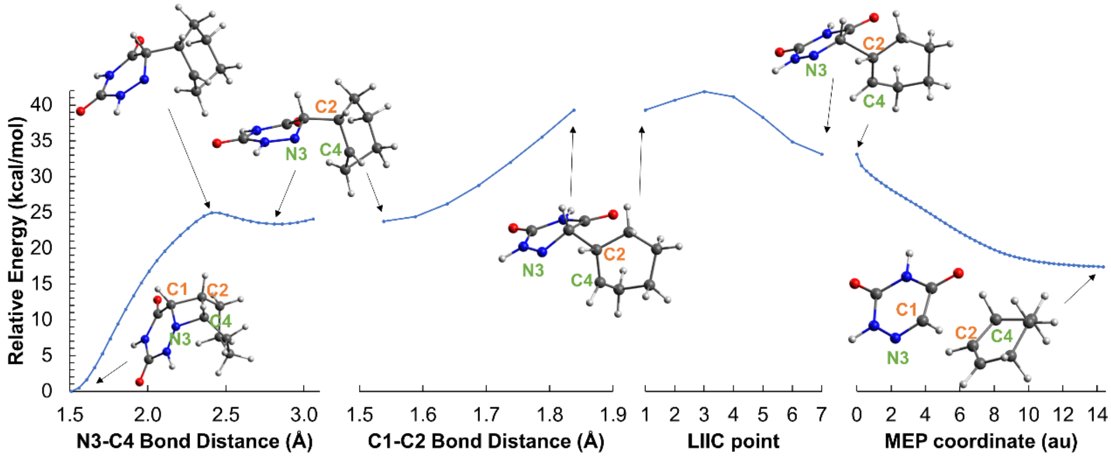

Figure S5. Opening of the azetidine ring of the *cis*-AZT-CH system in the gas phase initiated by the N_3-C_4 bond breaking. The reaction profiles correspond to the oxidized system with a net charge of +1 and doublet multiplicity, and have been obtained through relaxed scans of the N_3-C_4 and C_1-C_2 bond distances, linear interpolation of internal coordinates (LIIC), and MEP determinations. As indicated by the back arrows, the relaxed scan of the C_1-C_2 bond distance was initiated from the most stable structure that exhibit N_3-C_4 bond cleavage (2.808 Å), and performed freezing the N_3-C_4 bond distance at this value to avoid the return of the system to the reagents region. The pathway that connects the last MEP structure to the products minimum shown in Figure 7 of the main text has not been computed.

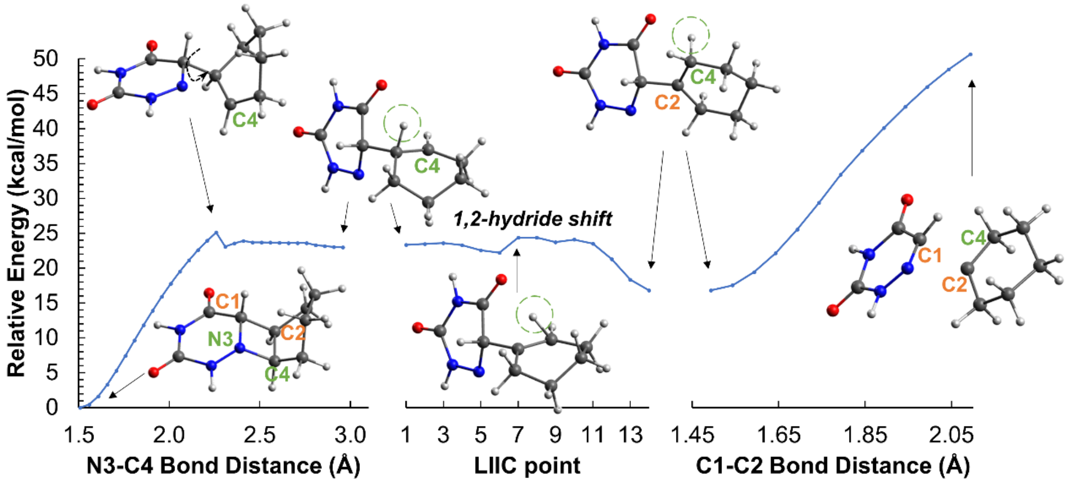

Figure S6. Opening of the azetidine ring of the *trans*-AZT-CH system in the gas phase initiated by the N_3-C_4 bond breaking. The reaction profiles correspond to the oxidized system with a net charge of +1 and doublet multiplicity, and have been obtained through relaxed scans of the N_3-C_4 and C_1-C_2 bond distances and linear interpolation of internal coordinates (LIIC) between relevant structures. LIIC point number 14 has been optimized without any constraint. The hydrogen atom that undergoes the 1,2-hydride shift is highlighted with green dashed circles.

Table S1. Energy differences between products and reactants (ΔE, ΔE_0, ΔG) and activation energies ($\Delta E^{‡}$, $\Delta E_0^{‡}$, $\Delta G^{‡}$) for the photocycloreversion of the neutral *cis*- and *trans*-AZT-CH isomers, in the gas phase and in solution. Energies are given in kcal mol^{-1}.

**Methodology**	***cis*-AZT-CH**		***trans*-AZT-CH**	
	ΔE	$\Delta E^{‡}$	ΔE	$\Delta E^{‡}$
M06-2X	-10.33	65.01	-11.48	64.83
PCM-M06-2X	-9.94	64.30	-10.29	64.67
	ΔE_0	$\Delta E_0^{‡}$	ΔE_0	$\Delta E_0^{‡}$
M06-2X	-13.07	61.34	-14.64	61.01
PCM-M06-2X	-12.68	60.63	-13.45	60.85
	ΔG	$\Delta G^{‡}$	ΔG	$\Delta G^{‡}$
M06-2X	-15.88	60.54	-19.62	60.10
PCM-M06-2X	-15.49	59.83	-18.43	59.93

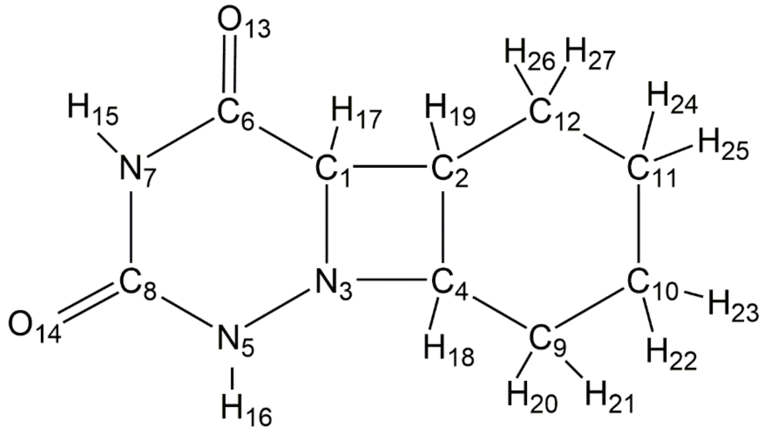

Figure S7. Atom labels of the AZT-CH system.

Table S2. Mulliken atomic spin densities for the relevant points on the ring opening reaction of *cis*- and *trans*-AZT-CH isomers of the anionic system. Only the most relevant positive atomic densities are shown, the total spin density is 1.

	Atom	Mulliken atomic spin density
cis-AZT-CH$^{\cdot -}$		
Reactants	C_6	1.046
TS1 (C_1-C_2 breaking)	C_2	0.508
	C_6	0.311
TS2 (N_3-C_4 breaking)	C_2	0.932
Products	N_3	0.475
	C_6	0.341
trans-AZT-CH$^{\cdot -}$		
Reactants	C_6	1.045
TS1 (C_1-C_2 breaking)	C_2	0. 503
	C_6	0.307
TS2 (N_3-C_4 breaking)	C_2	0.913
Products	N_3	0.518
	C_6	0.197

Table S3. Mulliken atomic spin densities for the relevant points on the ring opening reaction of *cis*- and *trans*-AZT-CH isomers of the cationic system. Only the most relevant positive atomic densities are shown, the total spin density is 1.

	Atom	Mulliken atomic spin density
cis-AZT-CH$^{\cdot +}$		
Reactants	N_3	0.493
	N_5	0.371
TS1 (C_1-C_2 breaking)	C_2	0.718
TS2 (N_3-C_4 breaking)	C_2	0.654
	C_4	0.261
Products	C_4	0.504
	C_2	0.460
trans-AZT-CH$^{\cdot +}$		
Reactants	N_3	0.477
	N_5	0.394
TS1 (C_1-C_2 breaking)	C_2	0.781
TS2 (N_3-C_4 breaking)	C_2	0.654
	C_4	0.261
Products	C_4	0.504
	C_2	0.460

Table S4. Mulliken atomic charges for each atom of the *cis*-AZT-CH anion and sum of the charges for both fragments, *i.e.* 6-azauracil and cyclohexene in the reactants, transition states and products.

***cis*-AZT-CH$^{\cdot -}$ Mulliken charges**				
Atom label	reactants	TS1 (C_1-C_2 breaking)	TS2 (N_3-C_4 breaking)	products
N_7	-0.40793	-0.426166	-0.519578	-0.4721
C_8	0.546756	0.532996	0.602183	0.570944
N_5	-0.53812	-0.61287	-0.799852	-0.678128
N_3	0.092886	0.195189	0.014692	0.059472
C_1	-0.002999	-0.623092	-0.441099	-0.23582
C_6	-0.558744	0.21513	0.461627	0.264927
C_4	-0.957931	-0.53682	-0.209638	-0.694023
C_2	0.599589	0.321088	0.224291	0.599045
C_9	0.265884	-0.004313	-0.52008	0.064922
C_{10}	-0.486388	-0.371836	-0.259374	-0.628249
C_{11}	-0.174072	-0.316049	-0.275613	-0.366059
C_{12}	-0.48177	-0.504056	-0.43204	-0.609291
O_{13}	-0.696397	-0.700947	-0.766047	-0.759239
O_{14}	-0.659975	-0.620639	-0.6275	-0.622852
H_{15}	0.33682	0.37397	0.37155	0.368069
H_{16}	0.382534	0.379746	0.371771	0.37285
H_{17}	0.052766	0.115808	0.113073	0.132041
H_{18}	0.114568	0.104325	0.18175	0.130577
H_{19}	0.184619	0.150013	0.166036	0.171298
H_{20}	0.158891	0.169344	0.191663	0.177921
H_{21}	0.212108	0.1973	0.168017	0.183504
H_{22}	0.156221	0.16208	0.156123	0.16793
H_{23}	0.149978	0.151373	0.152195	0.151866
H_{24}	0.153434	0.155956	0.163935	0.152183
H_{25}	0.229187	0.191668	0.174509	0.163968
H_{26}	0.17721	0.152679	0.164848	0.166449
H_{27}	0.150875	0.148122	0.17256	0.167794
Sum of charges				
6-azauracil	-1.452403	-1.170875	-1.21918	-0.999836
cyclohexene	0.452403	0.170874	0.219182	-0.000165

Table S5. Mulliken atomic charges for each atom of the *trans*-AZT-CH anion and sum of the charges for both fragments, *i.e.* 6-azauracil and cyclohexene in the reactants, transition states and products.

***trans*-AZT-CH$^{\cdot -}$ Mulliken charges**				
Atom label	reactants	TS1 (C_1-C_2 breaking)	TS2 (N_3-C_4 breaking)	products
N_7	-0.442959	-0.496374	-0.510062	-0.504573
C_8	0.606781	0.584966	0.622982	0.627613
N_5	-0.575865	-0.611735	-0.754204	-0.706638
N_3	-0.071587	-0.019181	0.009340	-0.030008
C_1	-0.174724	-0.60303	-0.491493	-0.427598
C_6	-0.265601	0.415713	0.501541	0.427817
C_4	-0.053859	-0.211234	-0.435325	-0.480414
C_2	0.107837	0.157514	0.285642	0.161306
C_9	-0.35767	-0.359429	-0.309589	-0.263457
C_{10}	-0.287305	-0.266068	-0.260421	-0.227971
C_{11}	-0.371624	-0.396714	-0.405521	-0.444022
C_{12}	-0.259765	-0.253305	-0.359241	-0.253722
O_{13}	-0.701366	-0.722613	-0.767682	-0.777474
O_{14}	-0.648686	-0.631765	-0.638811	-0.622323
H_{15}	0.361485	0.370664	0.371818	0.367892
H_{16}	0.366445	0.366716	0.374301	0.37041
H_{17}	0.151495	0.139762	0.117509	0.125901
H_{18}	0.160609	0.143606	0.179917	0.152798
H_{19}	0.183802	0.171576	0.168120	0.171727
H_{20}	0.160796	0.151601	0.135683	0.179798
H_{21}	0.160748	0.160022	0.177178	0.170584
H_{22}	0.161639	0.159583	0.160057	0.155552
H_{23}	0.159946	0.162146	0.176904	0.173089
H_{24}	0.162563	0.14331	0.155306	0.155575
H_{25}	0.151053	0.147947	0.159641	0.159334
H_{26}	0.15902	0.147313	0.159079	0.155225
H_{27}	0.156792	0.14901	0.177331	0.183579
Sum of charges				
6-azauracil	-1.394582	-1.206877	-1.164761	-1.148981
cyclohexene	0.394582	0.206878	0.164761	0.148981

Table S6. Mulliken atomic charges for each atom of the *cis*-AZT-CH cation and sum of the charges for both fragments, *i.e.* 6-azauracil and cyclohexene, in the reactants, transition states and products.

***cis*-AZT-CH$^{\cdot+}$ Mulliken charges**				
Atom label	reactants	TS1 (C_1-C_2 breaking)	TS2 (N_3-C_4 breaking)	products
N_7	-0.345335	-0.436147	-0.537438	-0.499371
C_8	0.511443	0.510817	0.557336	0.646715
N_5	-0.622271	-0.754911	-0.565795	-0.445706
N_3	0.46334	0.496111	0.173842	0.165842
C_1	-0.162679	-0.059295	-0.096237	-0.115995
C_6	0.030099	0.206077	0.434451	0.280444
C_4	-0.143504	0.050584	0.338267	0.297866
C_2	0.160071	-0.05393	0.003559	0.038879
C_9	-0.450184	-0.47045	-0.553115	-0.492401
C_{10}	-0.229649	-0.25486	-0.322484	-0.402875
C_{11}	-0.227108	-0.265786	-0.290608	-0.322435
C_{12}	-0.543785	-0.47937	-0.504429	-0.432112
O_{13}	-0.344887	-0.4292	-0.490373	-0.61496
O_{14}	-0.398531	-0.43777	-0.447874	-0.401583
H_{15}	0.428189	0.432837	0.423104	0.405814
H_{16}	0.472858	0.470263	0.456847	0.422899
H_{17}	0.307746	0.272282	0.195611	0.247315
H_{18}	0.255304	0.280832	0.283057	0.236254
H_{19}	0.245465	0.259356	0.249048	0.213298
H_{20}	0.215654	0.226297	0.168426	0.250438
H_{21}	0.205847	0.18913	0.262763	0.228799
H_{22}	0.188421	0.181125	0.189225	0.19653
H_{23}	0.199197	0.203372	0.194821	0.201241
H_{24}	0.199048	0.205729	0.209912	0.21168
H_{25}	0.18549	0.215367	0.209635	0.204944
H_{26}	0.203815	0.213835	0.238730	0.247293
H_{27}	0.195947	0.227704	0.219720	0.231186
Sum of charges				
6-azauracil	0.339972	0.271064	0.103474	0.091414
cyclohexene	0.660029	0.728935	0.896527	0.908585

Table S7. Mulliken atomic charges for each atom of the *trans*-AZT-CH cation and sum of the charges for both fragments, *i.e.* 6-azauracil and cyclohexene, in the reactants, transition states and products.

***trans*-AZT-CH$^{\cdot+}$ Mulliken charges**				
Atom label	reactants	TS1 (C_1-C_2 breaking)	TS2 (N_3-C_4 breaking)	products
N_7	-0.449123	-0.500129	-0.537438	-0.499371
C_8	0.621811	0.638032	0.557336	0.646715
N_5	-0.45979	-0.62183	-0.565795	-0.445706
N_3	0.409407	0.602685	0.173842	0.165842
C_1	-0.663745	-0.32088	-0.096237	-0.115995
C_6	0.334284	0.445491	0.434451	0.280444
C_4	-0.251633	-0.517712	0.338267	0.297866
C_2	0.309313	0.293601	0.003559	0.038879
C_9	-0.359083	-0.265778	-0.553115	-0.492401
C_{10}	-0.232261	-0.227637	-0.322484	-0.402875
C_{11}	-0.352939	-0.34769	-0.290608	-0.322435
C_{12}	-0.388526	-0.527174	-0.504429	-0.432112
O_{13}	-0.390211	-0.434667	-0.490373	-0.61496
O_{14}	-0.410015	-0.455963	-0.447874	-0.401583
H_{15}	0.44212	0.44212	0.423104	0.405814
H_{16}	0.461716	0.447238	0.456847	0.422899
H_{17}	0.317764	0.211997	0.195611	0.247315
H_{18}	0.257593	0.231948	0.283057	0.236254
H_{19}	0.249419	0.261657	0.249048	0.213298
H_{20}	0.197987	0.200163	0.168426	0.250438
H_{21}	0.202443	0.212426	0.262763	0.228799
H_{22}	0.198352	0.210444	0.189225	0.19653
H_{23}	0.198661	0.193621	0.194821	0.201241
H_{24}	0.178058	0.191654	0.209912	0.21168
H_{25}	0.187292	0.198464	0.209635	0.204944
H_{26}	0.198202	0.22722	0.238730	0.247293
H_{27}	0.192906	0.210699	0.219720	0.231186
Sum of charges				
6-azauracil	0.214218	0.454094	0.103474	0.091414
cyclohexene	0.785784	0.545906	0.896527	0.908585
